# Epigenetic implications in the pathogenesis of corticotroph tumors

**DOI:** 10.1007/s11102-025-01522-3

**Published:** 2025-04-21

**Authors:** Ticiana Paes, Leo J. Hofland, Anand M. Iyer, Richard A. Feelders

**Affiliations:** 1 Department of Internal Medicine, Division of Endocrinology, Erasmus Medical Center, Erasmus MC Cancer Institute, Dr. Molewaterplein 40, Rotterdam, GD 3015 The Netherlands; 2 Department of Internal Medicine, Roger Williams Medical Center, Providence, RI USA; 3 Division of Endocrinology, Diabetes and Metabolism, New York University Langone Medical Center, New York, NY 10016 USA

**Keywords:** Epigenetic, DNA methylation, Cushing disease, Corticotroph tumors

## Abstract

Non-mutational epigenetic reprogramming is considered an important enabling characteristic of neoplasia. Corticotroph tumors and other subtypes of pituitary tumors are characterized by distinct epigenetic profiles. The DNA methylation profile is consistent with disease-specific gene expression, which highlights the importance of epigenetic changes in tumor formation and progression. Elucidating the epigenetic changes underlying tumorigenesis plays an important role in understanding the molecular pathogenesis of corticotroph tumors and may ultimately contribute to improving tumor-specific treatment. Here, we provide an overview of the epigenetic landscape of corticotroph tumors. We also review the role of epigenetics in silencing the expression of tumor suppressor genes and promoting oncogenes expression, which could potentially be involved in the pathogenesis of corticotroph tumors. We briefly discuss microRNAs and epigenetic aspects of POMC regulation. Lastly, since the epigenetic changes are reversible, we discuss drugs that target epigenetic modifiers that could potentially be used in the arsenal of Cushing’s disease treatment modalities.

## Introduction

Tumorigenesis often starts with a driver mutation that gives rise to cell proliferation, called the breakthrough phase [1]. As the tumor progresses, the cells acquire more genetic and/or epigenetic alterations that contribute to dysregulation of growth-related pathways [2]. 

ACTH-secreting pituitary tumors are rare, occurring mostly in sporadic form, uncommonly being part of genetic familiar syndromes. While the understanding of their pathogenesis is limited, recent research has unraveled some driver somatic mutations responsible for the pathological process [3–5]. 

**Fig. 1:**
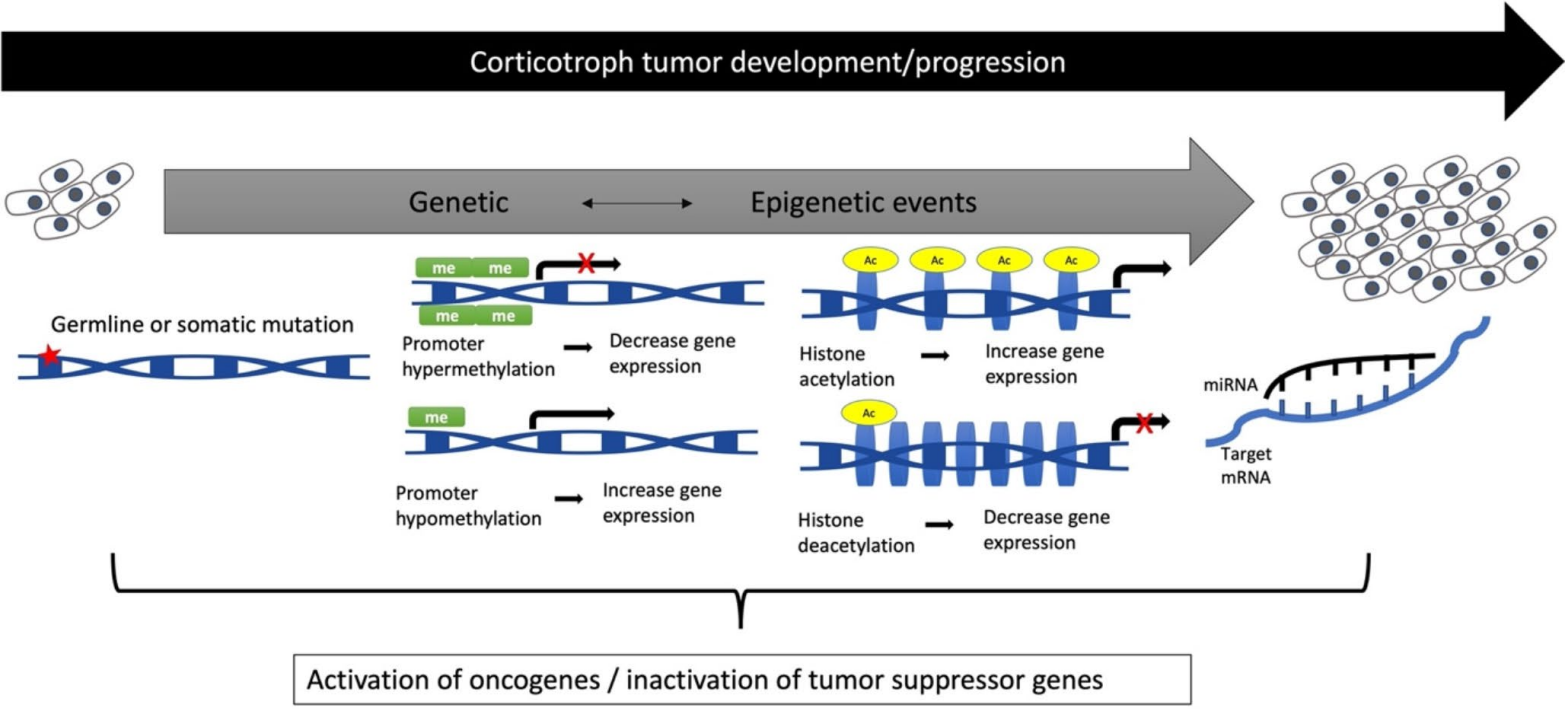
Corticotroph tumor pathogenesis. The interplay between genetic and epigenetic factors may influence the expression of oncogenes and/or tumor suppressor genes, thereby contributing to the formation and progression of corticotroph tumors. Epigenetic changes, including histone modifications, DNA methylation, and miRNA may impact the transcription of genes potentially involved in the tumorigenesis process. Ac: acetylation, me: methylation, miRNA: microRNA

Recurrent mutations are not the only biological process affecting corticotroph tumors. An altered epigenetic landscape can potentially be involved as driving force in tumorigenesis as well, such as impeding cell death or stimulating proliferative signaling. The interaction between genetic and epigenetic alterations may impact tumor formation and progression [2, 6] (Fig. 1). Therefore, the elucidation of the epigenetic changes underlying tumorigenesis plays an important role in the comprehension of the molecular pathogenesis of ACTH-secreting tumors and may ultimately contribute to improve tumor-specific treatment.

In the present article, we provide an overview of the epigenetic mechanisms and candidate genes potentially involved in the pathogenesis of corticotroph tumors, as well as their potential implications for treatment.

## Epigenetic modifications

Epigenetics is a generic term used to describe modifications that affect gene expression without alterations in the DNA code. Such modifications are reversible and heritable [7]. Epigenetic modifications are essential to establish an appropriate regulation of gene expression for a normal cellular process. The most described epigenetic changes are DNA methylation and histone acetylation, which generally interact with each other to interfere with the chromatin state, turning on or off gene expression [8]. Non-coding RNAs, such as microRNAs (miRNAs), are another epigenetic mechanism that influences gene expression by inducing RNA degradation or blocking its translation to protein [9] (Fig. 1).

A strict balance among the epigenetic mechanisms is essential to avoid aberrant gene expression leading to diseases. Nevertheless, an environmental, metabolic or genetic insult may induce epigenetic reprogramming, contributing to tumor formation and progression [10]. 

## Epigenetic landscape of corticotroph tumors

**Fig. 2:**
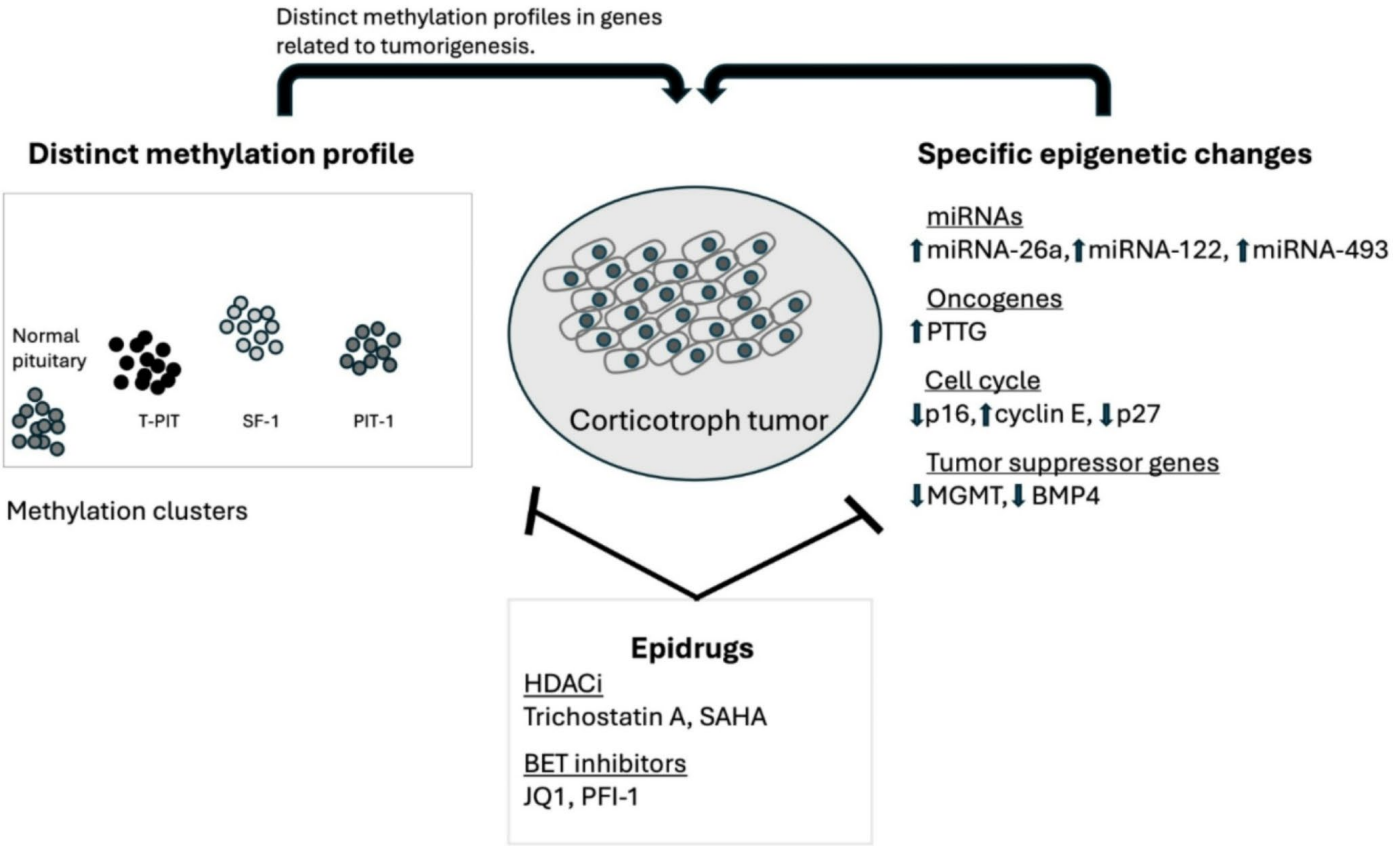
Epigenetic changes potentially be involved in corticotroph tumorigenesis. Genome-wide studies have shown distinct methylation profiles according to pituitary tumor subtype. Methylation changes associated with corticotroph tumors can influence the dysregulation of growth-related pathways, contributing to tumorigenesis. Tumor suppressor genes and/or oncogenes can be epigenetically disrupted, contributing to corticotroph tumor initiation and/or progression. Finally, epigenetic changes are reversible processes that can be achieved by using epidrugs. Epidrugs can be potent anti-tumor compounds and may become potential drugs for CD treatment. HDACi: Histone deacetylase inhibitor; SAHA: Suberoylanilide hydroxamic acid; BET: Bromo and extra-terminal domain

Genome-wide studies have shown distinct methylation profiles according to pituitary tumor subtype [4, 11–14]. They reflect adenohypophyseal cell lineages characterized by transcription factor coding genes as follows: PIT-1 (somatotroph, lactotroph and tyrotroph cell lineages) being hypomethylated; SF-1 enriched (gonadotroph cell lineage) being hypermethylated, and T-PIT enriched (corticotroph cell lineage) having intermediate methylation levels [14]. These distinct methylation profiles were observed in genes related to cell differentiation and tumorigenesis [14] (Table 1; Fig. 2).

**Table 1:** Genome-wide methylation studies on pituitary tumors including corticotroph tumors

Study	Year	*n*	Population	Method	Results	Interpretation
[11]	2012	32 PT 4 post-mortem normal pituitaries	7 GH, 6 CD, 6 PRL, 13 NFPT	- Illumina Infinium 27 K BeadArray - DNA Pyrosequencing analysis to validate bead-array analysis	- Subtype-specific methylation pattern was identified	- In corticotroph tumors, *KIAA0676* was hypermethylated gene relative to post-mortem normal pituitary.
[16]	2014	24 PT	17 NFPT, 1 silent ACTH, 5 GH, 1 CD	Illumina Infinium HumanMethylation450 BeadChip array	- In a comparison between invasive and non-invasive tumors, no significant differences in DNA methylation levels were detected - two methylation groups were identified, non-functional cluster composed of all NFPT (including 1 silent ACTH) and 1 CD tumor; and functional cluster with all 5 somatotroph tumors	- Corticotroph tumors, one CD and one silent ACTH tumors, clustered to the same group of non-functional tumors However, very limited patient number
[13]	2018	48 PT^‡^	17 GH; 13 ACTH (10 CD and 3 silent-ACTH); 18 NFPT	Illumina Infinium Human Methylation450 BeadChip array	- Three different methylation clusters according to tumor subtype (GH, ACTH and NFPT).	- Silent ACTH tumors clustered with CD tumors - *POMC* promoter hypomethylation leading to *POMC* overexpression was observed in corticotroph tumors
[4]	2019	134 PT^†^	35 ACTH, 29 gonadotroph, 8 null-cell, 23 GH, 16 PRL, 8 GH-PRL 6 TSH, 9 plurihormonal PIT1-positive	Illumina Infinium MethylationEPIC BeadChip	- Three methylation patterns were observed which were correlated with tumor type and secretion.	- Pangenomic classification of pituitary tumors was suggested, in which corticotroph tumors belong to three distinct groups: tumors with USP8 mutation, USP8 wild-type tumors with more aggressive behavior and silent ACTH tumors with gonadotroph trans-differentiation The possibility of “contamination” with non-neoplastic tissue is questioned [115]
[12]	2020	42 PT; 6 non-tumoral pituitaries	10 GH, 2 PRL, 4 TSH, 6 CD, 20 NFPT (including 3 silent ACTH)	Infinium Methylation BeadChip Array (EPIC)	- Three clusters were identified according to transcription factors, as follows, TPIT-driven ACTH, SF1-driven NF and PIT1-driven (GH-, PRL and TSH tumors)	- Silent corticotroph-ACTH tumors clustered separately with common features of both NF tumors and CD tumors. - Promoter hypomethylation leading to increased gene expression in ACTH tumors: AVPR1B, EPHA4, GRIN2B, GRIA4, miRNA592 and miRNA4796
[15]	2020	36 ACTH tumors	15 CD; 21 silent ACTH	850k or 450k Illumina arrays	- 3 clusters were identified: two distinct CD and silent ACTH cluster, and a third cluster that presented varied clinical manifestation	- DNA methylation separate CD from silent ACTH. - A potential “intermediate state” of CD and silent ACTH, with a functional status of subclinical Cushing, was suggested based on DNA methylation data.
[14]	2021	177 PT*; 86 PT (validated cohort) 20 non-tumoral pituitaries	87 FPT and 90 NFPT**	- Illumina Human Methylation BeadChip arrays (HM450K) [16, 116, 117] - Methylation BeadChip (EPIC) array	- Methylation profiles were identified reflecting cell lineage and functional status. - TPIT cluster: 21 CD, 1 silent ACTH, 1 gonadotroph, 1 null cell, 5 NFPT unknown subtype	- TPIT cluster: intermediately methylated - Silent ACTH and CD shared a common methylation profile. - Part of gonadotroph and ACTH tumors shared methylation features. - Very limited number of patients - TPIT-related probe was hypomethylated and associated with a greater TPIT expression
[17]	2023	77 PT	18 GH, 14 CD; 45 NFPT (5 silent ACTH tumors)	Infinium Methylation EPIC BeadChip Array	-3 major clusters were identified: **A**. functioning tumors with 2 distinguished branches of CD and GH tumors; **B**. nonfunctioning tumors (including 2 silent ACTH); **C**. mixed cluster with CD (USP8 mutated mostly) and nonfunctioning tumors (including 3 silent ACTH)	- CD tumors clustered into two distinct methylation profile (cluster A and C). - Most of USP8 mutated tumors (80%) grouped in cluster C together with NF and silent ACTH tumors. - Silent ACTH shared methylation profile with both CD and NFPT.

PT: pituitary tumors. GH: somatotroph tumors. PRL: prolactinomas. ACTH: corticotroph tumors. CD: Cushing’s disease tumors; FPT: functioning-pituitary tumors; NFPT: non-functioning pituitary tumors

‡ Methylome analysis was performed in 37 pituitary tumors

†Methylome analysis was performed in 86 pituitary tumors

* The total cohort consisted of publicly available DNA methylation data from three independent studies [16, 116, 117] and the cohort at the Hermelin Brain Tumor Center (HBTC) of the Henry Ford Health System (HFHS) (Detroit, MI, USA) (*n* = 23)

** Functional pituitary tumors (FPT): 21 ACTH (CD); 41 GH; 2 GH/PRL; 1 GH/TSH; 2 GH/PRL/TSH; 11 TSH; 9 PRL; non-functional pituitary tumors (NFPT): 1 silent ACTH; 62 LH and/or FSH; 2 LH/FSH/TSH; 8 null cells; 17 unknown

In a study involving only corticotroph tumors, 15 CD and 21 silent ACTH, the genome-wide methylation profile was able to differentiate CD tumors from silent corticotroph [15]. In addition, a third methylation class was identified in which most of the subclinical pituitary Cushing cases were included, some cases of silent ACTH, and only one case of CD [15] (Table 1).

Regarding the silent corticotroph tumors, genome-wide studies question their methylation pattern. Some studies reported that silent corticotroph tumors share a similar methylation profile with Cushing’s disease (CD) tumors and are grouped in the same hierarchical cluster, reflecting the same cell lineage [13, 14, 16]. On the other hand, other studies reported silent corticotroph tumors sharing methylation signatures with both non-functioning pituitary tumors and CD tumors [12, 17] (Table 1). While these studies offer insights into the methylation pattern of silent corticotroph tumors, the small number of tumors included (ranging from 1 to 5) may be considered a limitation in drawing a definitive conclusion yet.

A multiplatform study, including genomic, epigenomic and transcriptomic analyses, also revealed characteristic methylation patterns associated with tumor subtypes, comprising corticotroph tumors, among others. The DNA methylation profile was consistent with disease-specific gene expression, which highlights the importance of epigenetic changes in tumor formation and progression [13]. 

The modification in DNA methylation in pituitary tumors may partially be explained by DNA methyltransferase (DNMT) enzyme expression levels. Overexpression of DNMTs was found in some pituitary tumors, including corticotroph tumors, and was associated with hypermethylation of tumor suppression genes [18]. Conversely, in the mouse corticotroph tumor cell line AtT20 dexamethasone dose-dependent downregulation of DNMT1 has been described, which could lead to hypomethylation events [19]. Neou et al. suggested the participation of the TET2 enzyme, involved in the active demethylation process, in the hypomethylated pattern of PIT1-driven tumors [4]. 

Taken together, these pieces of evidence support the concept that a distinct epigenetic profile characterizes each subtype of pituitary tumor, including corticotroph tumors.

## Epigenetic mechanisms of POMC regulation

Physiologically, POMC synthesis in corticotroph cells is stimulated by CRH and inhibited by glucocorticoids (GC) [20]. In Cushing’s disease, there is a relative loss of sensitivity to GC´s negative feedback on POMC expression. Accordingly, the administration of dexamethasone (DEX), used as a diagnostic test, results in an insufficient decrease in cortisol levels in most patients [21]. This GC resistance may be explained by overexpression of HSP90 chaperone, impairing GC activity [22], and 11β-hydroxysteroid dehydrogenase type 2, inactivating cortisol [23, 24]. 

Another mechanism is the loss of histone deacetylase type 2 (HDAC2) or BRG1 expression, ATPase component of the SWI/SNF complex, which has been observed in half of studied corticotroph tumors. Normally, GC interacts with BRG1, HDAC2 and the nuclear receptor NGFI-B, establishing a trans-repression complex which, through a deacetylation process, decreases POMC transcription even in the presence of CRH. Therefore, the loss of critical components, such as BRG1 or HDAC2, of this trans-repression complex leads to less GC action in suppressing POMC expression accounting for GC resistance in some ACTH-secreting tumors [24, 25]. 

In addition to GC resistance contributing to POMC overexpression, epigenetic changes in the POMC promoter also regulate its expression in CD [26]. Transcriptomic differences between corticotroph tumors and non-functioning pituitary tumors revealed overexpression of POMC, which was attributed to the hypomethylation of the POMC promoter [13]. POMC promoter methylation is tissue-specific; it is heavily methylated in tissues without POMC expression, such as the adrenal gland. In contrast, it is weakly methylated in pituitary cells, even unmethylated in the AtT20 corticotroph cell line, leading to increased levels of POMC expression [26–28]. Interestingly, a second promoter identified in the human POMC gene expresses different levels of methylation, according to the type of corticotroph tumors. In silent corticotroph adenoma, the second promoter was highly methylated, while in CD tumors, it was highly unmethylated [29]. Epigenetic reprogramming of the POMC gene due to environmental cues has been observed [30, 31]. In an animal study, early-life stress exposure induced long-lasting hypomethylation in POMC regulatory regions resulting in increased pituitary POMC expression compared to control animals [27]. 

## Epigenetic changes in genes involved in corticotroph tumors pathogenesis

For pituitary tumor progression, a set of growth abilities must be acquired. This can be mediated by the involvement of tumor suppressor genes and/or oncogenes, which are epigenetically disrupted (Fig. 1).

### Pituitary tumor transforming gene (PTTG)

PTTG is a multifunctional protein involved in a variety of physiological processes, including DNA repair, cell cycle, apoptosis, and gene regulation. When highly expressed, PTTG plays a significant role as an oncogene in pituitary tumorigenesis [32–35]. 

High levels of PTTG, frequently found in pituitary tumors, were associated with pituitary tumor invasiveness and recurrence [36–38]. Higher expression of PTTG was also reported in corticotroph tumors, albeit with no association with clinicopathologic parameters, such as tumor recurrence, tumor size, symptom duration and invasiveness [39–41]. 

Evidence suggests that an epigenetic mechanism regulates PTTG expression levels, which may be cell-type specific [42]. In 293T human embryonic kidney epithelial cells, it has been shown that the use of HDAC inhibitor (HDACi), Trichostatin, caused an upregulation of PTTG transcription [43]. On the other hand, in the AtT20 corticotroph tumor cell line, the HDACi Trichostatin repressed the expression of PTTG mRNA in a dose-dependent manner [44]. This was confirmed in human colon carcinoma cells (HCT116) using another HDACi valproate [45]. Another mechanism may also be responsible for the abundance of PTTG in pituitary tumors. Recently, RWD-containing sumoylation enhancer (RSUME) protein was shown to be overexpressed in pituitary tumors, including corticotroph tumors. RSUME and PTTG are co-expressed, and a strong linear positive relationship was found between them. It was demonstrated that overexpression of RSUME may induce, by sumoylation process, PTTG protein stability responsible for increased PTTG half-life and, consequently, its overexpression [46]. 

Given its role in corticotroph tumor pathogenesis, PTTG overexpression may be a potential target for Cushing’s disease treatment with HDACi (Trichostatin) or SD1029, an anti-fungal agent and a potent JAK-STAT pathway inhibitor that targets PTTG to suppress AtT20 cell proliferation [47]. 

### Bone morphogenetic protein 4 (BMP4)

BMP4 is part of the transforming growth factor β (TGFβ) family. It is involved in pituitary organogenesis and diverse cellular functions, such as cell death, proliferation, and migration [48]. In the pituitary, BMP4 plays different roles depending on the cell type. It stimulates prolactin (PRL) secretion in lactotrophs cells, while it inhibits basal and CRH-induced ACTH production in corticotroph cells [48–50]. 

In addition, BMP4 has a tumor suppression activity. It was demonstrated that BMP4 reduces AtT20 cell line proliferation in in vitro and in in vivo models. BMP4-inhibition of the c-*myc* oncogenic activity may account for its antiproliferative effect [51]. Accordingly, reduced expression of BMP4 was found in ACTH-secreting tumors [51]. 

Epigenetic changes may explain reduced BMP4 expression in corticotroph tumors [52]. Histone modifications, but not methylation changes, were apparent in corticotroph tumors expressing reduction of BMP4 mRNA, and similar findings were also observed in other adenoma subtypes [52]. In the corticotroph tumor cell line AtT20, changes in methylation and histone modifications account for decreased expression of BMP4. Thus, incubation with DNA demethylating epidrug (zebularine) together with histone deacetylase inhibitor (Trichostatin A) was able to reverse the epigenetic modification, and a robust BMP4 re-expression was observed [52]. 

Hence, BMP4 seems to be an attractive target for CD treatment given its antiproliferative effect and its importance as a POMC negative regulator [50, 51]. Known drugs, such as retinoic acid and some somatostatin analogs, exert their pharmacologic effect through the BMP4 pathway. Retinoic acid induces BMP4 expression, while somatostatin analogs enhance BMP4 signaling in corticotroph tumor cells [49, 51, 53, 54]. 

### O6-methylguanine -DNA methyltransferase (MGMT)

MGMT is a DNA repair enzyme that prevents DNA strand breaks by removing the alkyl group from guanine residues [55]. The alkyl group is mutagenic and cytotoxic and can be triggered by carcinogens or alkylating drugs such as Temozolomide (TMZ) [56]. Thus, MGMT protects the cells from driver mutations that may elicit or contribute to tumorigeneses [57, 58]. 

Interestingly, pituitary tumors with high MGMT display a different set of activated genes than those with low MGMT expression. Upregulation of components linked to FGFR family and to signaling pathways, such as MAPK and PI2 K/Akt, were observed in a high MGMT expressing group, while genes involved in DNA repair systems and DNA damage response were the most upregulated in the low MGMT group [57]. 

In pituitary tumors, including invasive corticotroph tumors, low MGMT expression was also more frequent in aggressive compared to non-aggressive subtypes [59–64]. However, some studies failed to show MGMT association with invasiveness or other clinicopathological characteristics [65–68]. 

While epigenetic changes are the main reported mechanism responsible for silencing MGMT expression [56], a metanalysis comprising 2,943 patients with brain and non-brain tumors failed to demonstrate concordant results between MGMT protein expression and promoter hypermethylation [69]. In pituitary tumors, MGMT promoter methylation does not always correlate with MGMT protein expression, and other yet unraveled regulatory mechanisms may contribute as well [70–73]. 

## Epigenetic changes in genes involved in cell cycle related genes

Uncontrolled progression of cell cycle is part of the tumorigenesis process. Physiologically, the cell cycle comprises four successive events namely, gap 1 (G1), DNA synthesis phase (S), gap 2 (G2), and mitosis (M). These phases are coordinated by complexes containing cyclins and cyclin-dependent kinase (CDKs) and its negative regulator, the cyclin-dependent kinase inhibitor (CDKI) [74]. 

### p16

CDKN2A gene codes the protein p16 INK4a, an important inhibitor of CDK4/6-cyclin D complex. Once p16 INK4a is pathologically downregulated, the Rb protein remains inactivated and unable to halt the cell cycle, which may result in inappropriate cell division [75]. The loss of p16 INK4a function through CpG island methylation, is a frequent event in all types of sporadic pituitary tumors, and it is associated with larger tumors [42, 76, 77]. 

In corticotroph tumors both reduced and overexpression of p16 INK4a has been reported [40, 41, 78]. In a series of 31 corticotroph tumors, about half of the tumors exhibited p16 INK4a methylation with significant association with loss of protein expression in the majority of the cells within the specimen. In contrast, postmortem normal pituitary tissue failed to display p16 INK4a methylation. Interestingly, the nonadenomatous tissue from patients with proven CD, corticotroph hyperplasia, and apparently normal pituitary also exhibited p16 methylation, albeit in a smaller number of cells (10–20%) and without complete loss of the cognate protein. Possibly, p16 INK4a methylation is an initial event before tumor formation [79]. 

P16 INK4a seems to be overexpressed in functional ACTH-secreting tumors compared to silent corticotroph tumors. The excess of cortisol may play a role in increasing p16 INK4a expression, leading to cell cycle arrest. This could potentially explain why the majority of functional ACTH-secreting tumors are of a small size [78]. 

### p18

p18INK4c, is functionally connected to p16INK4a, with both reducing CDK4/CDK6 kinase activities. It has also been described to be downregulated in corticotroph tumors, which may contribute to the loss of proliferative control in these tumors [80, 81]. Hypermethylation of the CDKN2C gene may be involved in the loss of p18INK4c protein [82]. However, another study failed to detect CDKN2C promoter methylation in pituitary tumors [81]. 

### Rb

In the tumor suppression pathway, the activated Rb plays a critical role by blocking the cell cycle at G1 phase. Loss of Rb expression was reported in some human pituitary tumors, which was related to RB1 gene promoter methylation [83–85]. Interestingly, in a case report of two adjacent corticotroph tumors, one carcinoma and one adenoma, the absence of Rb expression was found only in the carcinoma specimen, indicating that the loss of Rb expression may imply a more aggressive behavior [86]. 

### Cyclin E and p27

In cell cycle progression, the active cyclinD-CDK4/6 complex drives cells to the G1/S transition phase, in which the cyclin E, CDK2, and p27kip1 (CDK inhibitor) are the key players. There is a mutual relationship between the tumor suppressor p27kip1 and cyclin E, both having an inhibitory effect on each other. P27kip1 inhibits the cyclin E / CDK2 complex, halting cell cycle progression. Cyclin E / CDK2 complex, in its turn, inactivates p27kip1 by phosphorylation, consequently accelerating the cell cycle. Therefore, an inverse correlation between cyclin E and p27kip1 would be expected in proliferating tumor cells, with an overexpression of cyclin E and a decreased p27kip1 expression [87]. 

Indeed, high cyclin E and low p27kip1 expression were reported in corticotroph tumors [87–90]. Overexpression of cyclin E is preferentially observed in corticotroph tumors compared to other lineages [88]. Low levels of p27kip1 expression and high levels of inactive p27kip1 (phosphorylated p27kip1) were found in ACTH-secreting tumors, which indicates reduced p27kip1 activity in controlling cell division [91]. Additionally, functional corticotroph tumors seem to exhibit lower expression of p27kip1 protein compared to non-secreting tumors [92]. 

One mechanism of p27kip1 reduced expression is a result of its increased degradation by the ubiquitin pathway [87]. Another mechanism is an epigenetic change involving histone methyltransferase (MLL) [85]. It has been shown that MLL can regulate the transcription of p27kip1 mRNA in the GH4C1 cell line [93]. However, this epigenetic mechanism has not yet been investigated in ACTH-secreting tumors.

At the S phase, the cells are already committed to continuing the cell cycle independently of growth factors. In mitosis, PTTG avoids abnormal chromosomal separation; its aberrant expression may contribute to genetic instability in pituitary tumor pathogenesis. (discussed in more detail in Sect. 4.1).

In conclusion, an epigenetic mechanism may account for aberration in the expression of cell cycle regulators leading to unrestrained cell proliferation. Nevertheless, the abnormal expression of cell cycle regulators can also be a consequence of an oncogenic pathway rather than a primary event in the pathogenesis of ACTH-secreting tumors [74, 94]. 

## The potential involvement of MicroRNA in corticotroph tumors pathogenesis

MicroRNAs (miRNA) represent a class of endogenous 18-22-nucleotide non-coding RNAs that function as small regulatory molecules involved in posttranscriptional gene repression [95–97]. MiRNAs act by binding to target mRNA, inducing its degradation or repressing its translation into protein, consequently interfering with gene expression. MiRNAs, through their target genes, are able to regulate some biological processes critical for tumor development, such as cell cycle and apoptosis.

Corticotroph tumors may display a dysregulated miRNAs profile with a potential role in its pathogenesis [98, 99]. miRNA-26a regulates cell cycle progression through silencing the expression of its target gene, the protein kinase Cδ (PRKCD), which was demonstrated using the AtT20 cell line. In human corticotroph tumors an inverse correlation was found between miRNA-26a and its target protein PRKCD, suggesting that the highly expressed miRNA-26a interferes in cell proliferation through repression of PRKCD protein [99]. In 2 ACTH-secreting carcinomas, miRNA-122 and miRNA-493 were overexpressed compared to adenomas, which may imply a potential role of these miRNAs in malignant transformation of the tumor [100]. miRNAs can also be involved in hormone secretion. Using AtT20 cell line, it has been demonstrated that miRNA375 negatively affects POMC expression in pituitary by targeting mitogen-activated protein kinase 8 (MAP3K8) [101]. 

USP8 mutation may be considered a separator of CD tumors [102, 103]. In transcriptomic analysis, USP8 mutated and wild-type tumors were segregated in a distinct gene cluster with distinct miRNA expression [4]. A recent study showed that 86 miRNAs were differentially expressed in USP8-mutated and wild-type corticotroph tumors [104]. These miRNAs were mainly related to ubiquitination process [104]. 

Several studies have provided evidence that dysregulated miRNA contributes to tumor development [98–100]. Nevertheless, a clear-cut picture of the mechanism through which altered miRNAs may underpin ACTH-secreting tumorigenesis is still missing.

## Epidrugs in the treatment of Cushing’s disease

The importance of a thorough understanding of the epigenetic changes in corticotroph tumors lies on the reversibility of this process achieved by the use of epidrugs [105]. Epidrugs act on families of epigenetic proteins, most commonly classified as readers, writers and erasers. Examples of a writer enzymes are DNA methyltransferase (DNMT), which adds a methyl group to cytosine in DNA, and histone acetyltransferase (HAT), which adds an acetyl group to a histone tail. Reader proteins, such as the bromodomain-containing family, recognize and bind to acetylated lysine residues. Eraser enzymes, such as histone deacetylase (HDAC), remove the epigenetic mark to ensure a dynamic and reversible process [105–107]. 

### Histone deacetylase inhibitor (HDACi)

HDAC inhibitors (HDACi) are potent antineoplastic compounds, especially in hematologic malignancies [108]. The effect of Trichostatin A an inhibitor of HDAC 1 and 2, on ACTH production and cell proliferation was demonstrated in AtT20 corticotroph tumor cells [44]. Trichostatin A incubation resulted in the reduction of POMC mRNA expression and the basal level of ACTH. It also induced cell cycle arrest and increased cell death in AtT20 cells. Presumably, the mechanism of Trichostatin A action is through its effect on reducing the PTTG oncogene expression [44]. 

Another HDAC inhibitor investigated in AtT20 tumor cells and in human corticotroph tumors is Suberoylanilide hydroxamic acid (SAHA, vorinostat), a pan-HDACi. Similar to Trichostatin A, SAHA was able to decrease the AtT20 viability by promoting cell death [109]. Additionally, SAHA decreased POMC transcription and consequently ACTH levels. As expected, the anti-proliferative effect and also the inhibition of ACTH secretion by SAHA was confirmed in primary cultures of ACTH-secreting tumors. Moreover, the anti-tumor action of SAHA proved to be effective in an animal model using athymic nude mouse AtT20 xenograft, significantly reducing tumor volume and ACTH secretion. Its mechanism of action was evaluated using large-scale gene expression analysis, which reveal that SAHA mediates the expression of genes related to apoptosis and nuclear receptors. Accordingly, POMC transcription was affected by downregulation of the nuclear receptor LXRα [109]. 

### Bromo and extra-terminal domain (BET) inhibitors

BET is a family of proteins functioning as an acetyllysine reader. It has the ability to recognize and bind to acetylated lysine residues in histones which serve as a scaffold for the transcription machinery, facilitating gene transcription. BET inhibitors are a new class of epidrugs which target bromodomain in the BET proteins thereby regulating gene expression. Their efficacy as an antineoplastic agent has been established in several human malignancies [110, 111]. 

Recently, the anti-proliferative effects of two BET inhibitors, JQ1 and PFI-1, were demonstrated in AtT20 corticotroph tumor cells [112]. JQ1 was a more potent inhibitor of cell growth than PFI-1. Its mechanism of action involves the regulation of apoptosis related genes, consequently inducing cell death. In addition, JQ1 was able to regulate POMC expression, hence interfering with ACTH secretion by AtT20 cells [112]. 

Epidrugs can be potent anti-tumor compounds and may become potential drugs for CD treatment. They can be used as an alternative or in combination with well-known drugs in a multi-target action. A drawback of the epigenetic-based therapy is its broad range of action, allowing off-target effects. Thus, advances in this therapeutic modality with the development of new compounds are underway to improve tumor-specific management [113, 114]. 

## Conclusion and outlook

The simplistic view that tumor pathogenesis is mainly related to genetic alterations has been broadening with growing knowledge of how epigenetic mechanisms regulate oncogene and/or tumor suppressor gene expression. The evidence of discrepant patterns of methylation in corticotroph tumors suggests a role in tumor initiation and/or progression. Since epigenetic changes are reversible, drugs targeting epigenetic modifiers could potentially be used in the arsenal of Cushing’s disease treatment (Fig. 2).

In conclusion, epigenetic modifications are elaborately controlled regulatory processes of gene expression. The alteration in this fine-tuned mechanism is part of corticotroph tumors’ ability to progress and adapt to the microenvironment. Research encompassing whole-exome sequencing (WES), methylome, and RNAseq analysis in combination with detailed clinicopathological data, focusing specifically on corticotroph tumors, can shed light on the complexity of their pathogenesis.

## Data Availability

No datasets were generated or analysed during the current study.
